# Supplementary material for: Aurora B controls anaphase onset and error-free chromosome segregation in trypanosomes
Source: J Cell Biol. 2024 Aug 28;223(11):e202401169. doi: 10.1083/jcb.202401169 (PMC11354203; doi:10.1083/jcb.202401169)
Supplement: SourceData F3 — is the source file for Fig. 3. [file JCB_202401169_SourceDataF3.pdf]

D

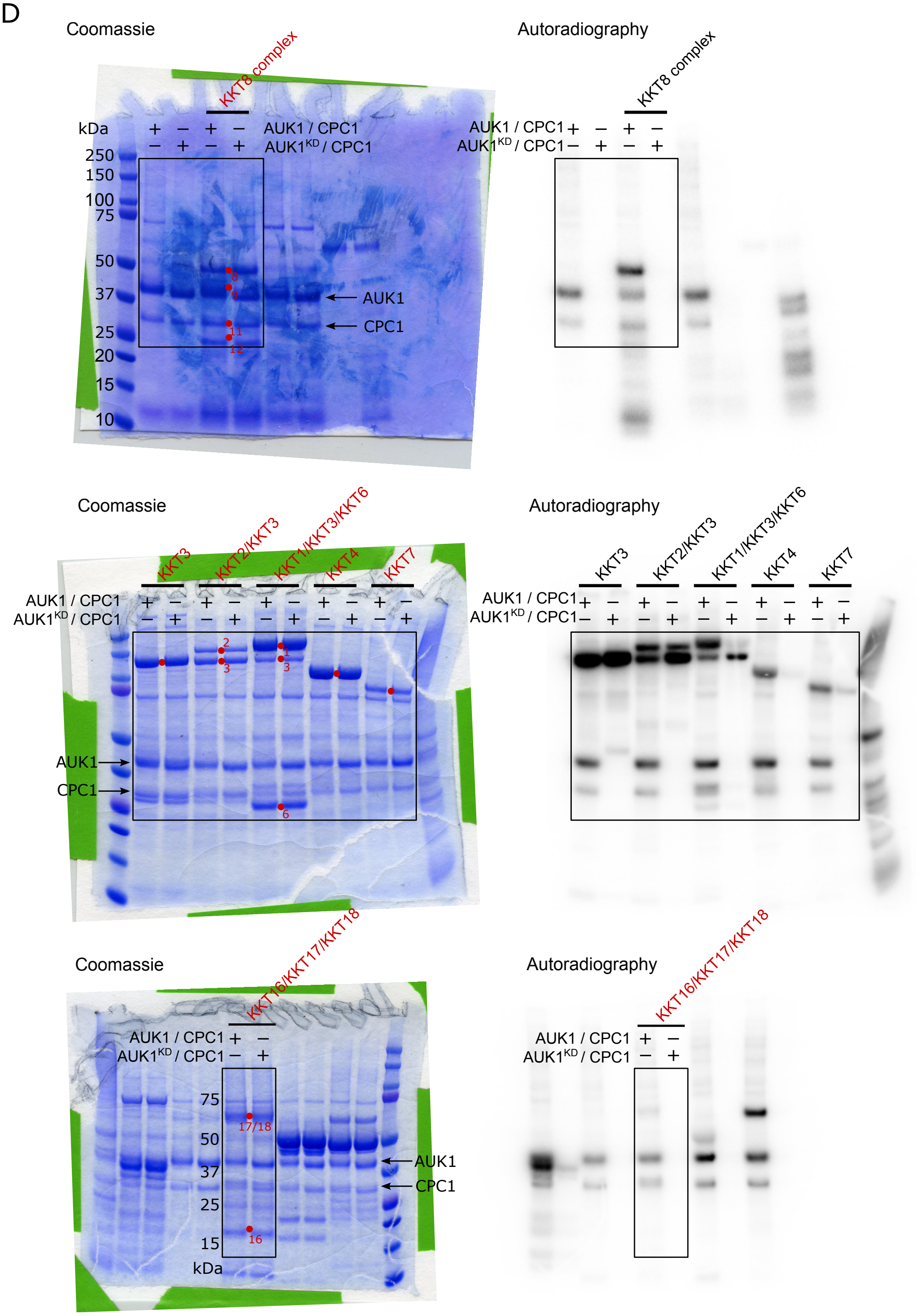
Coomassie
Autoradiography
KKT8 complex
kDa
AUK1 / CPC1
AUK1KD / CPC1
250
150
100
75
50
37
25
20
15
10
AUK1
CPC1
KKT3
KKT2/KKT3
KKT1/KKT3/KKT6
KKT4
KKT7
KKT16/KKT17/KKT18
17/18
16

E

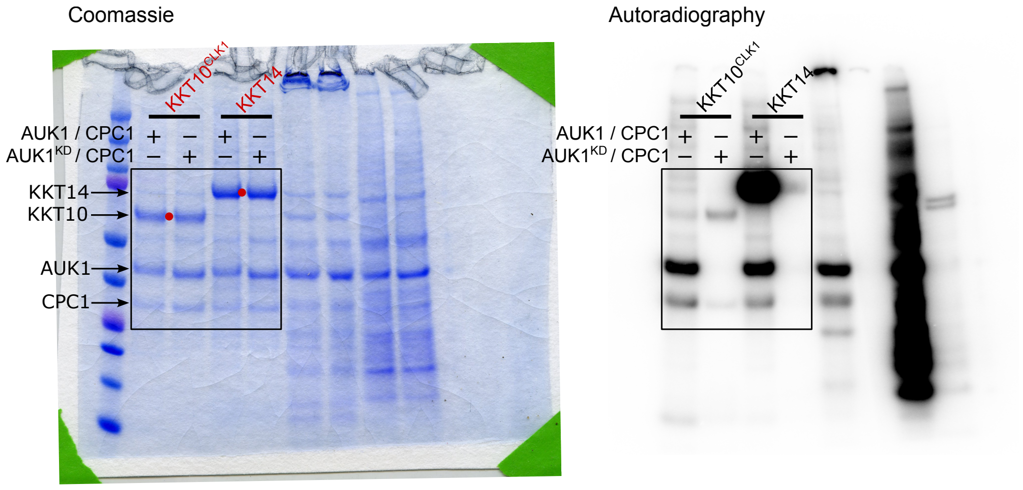
Coomassie
Autoradiography
KKT10CLK1
KKT14
AUK1 / CPC1
AUK1KD / CPC1
KKT14
KKT10
AUK1
CPC1
