# Supplementary figures and images for: Aurora B controls anaphase onset and error-free chromosome segregation in trypanosomes
Source: J Cell Biol. 2024 Aug 28;223(11):e202401169. doi: 10.1083/jcb.202401169 (PMC11354203; doi:10.1083/jcb.202401169)

A

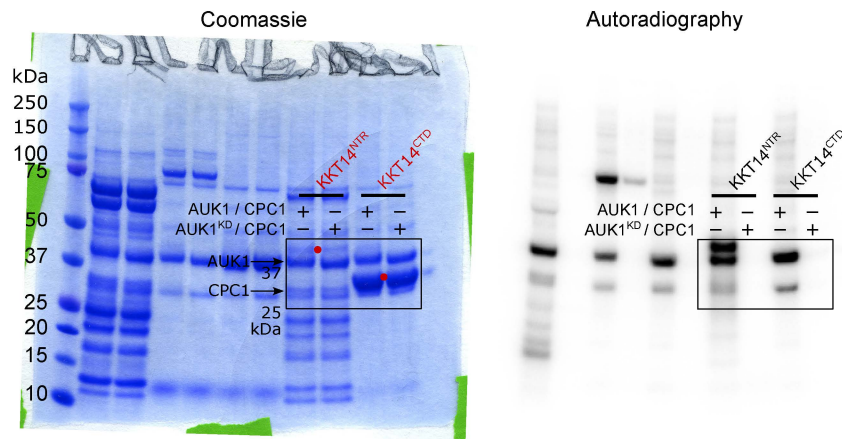

B

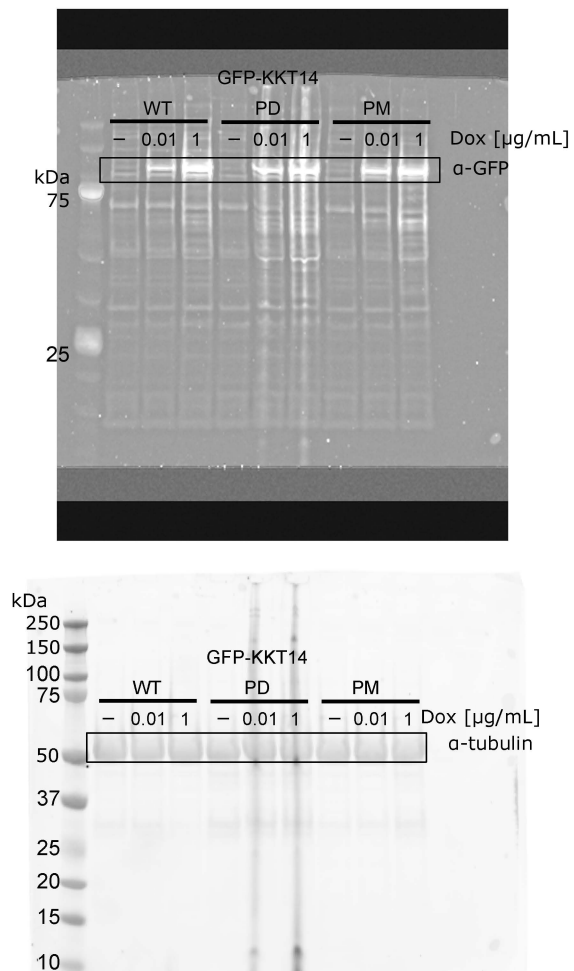

Supplement: SourceData F5 — is the source file for Fig. 5. [file JCB_202401169_SourceDataF5.pdf]

B

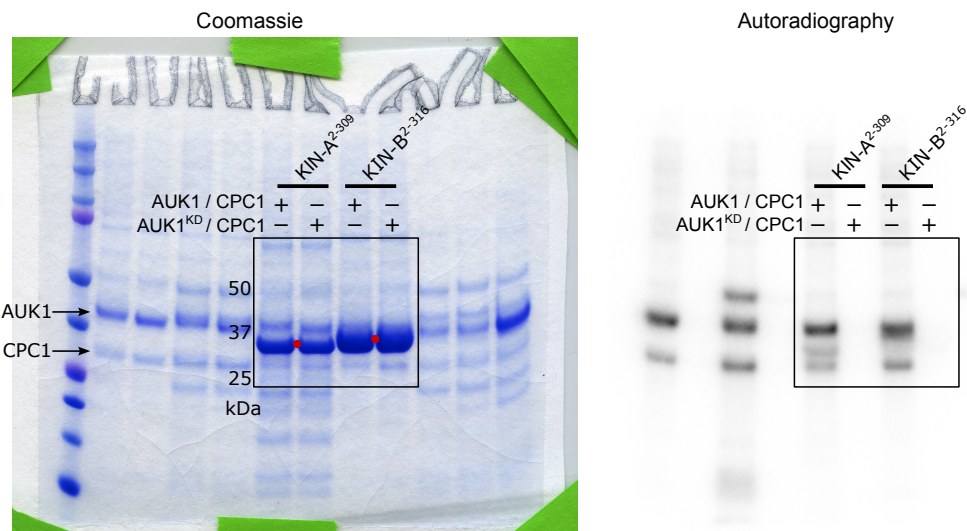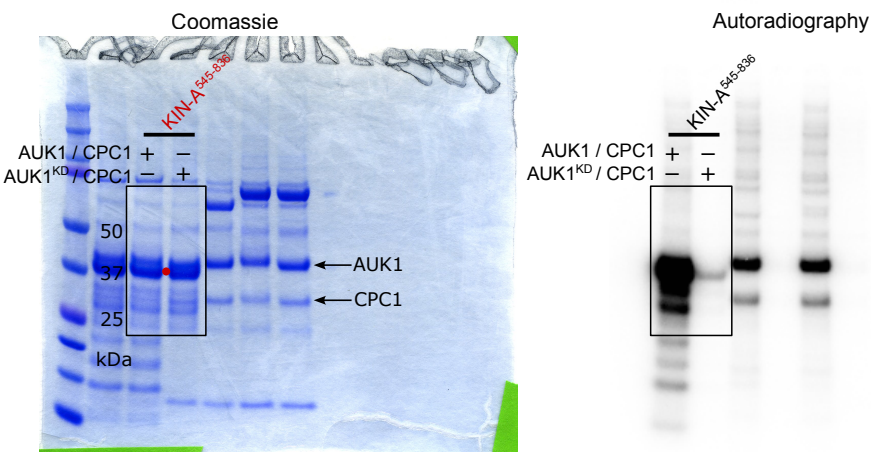

C

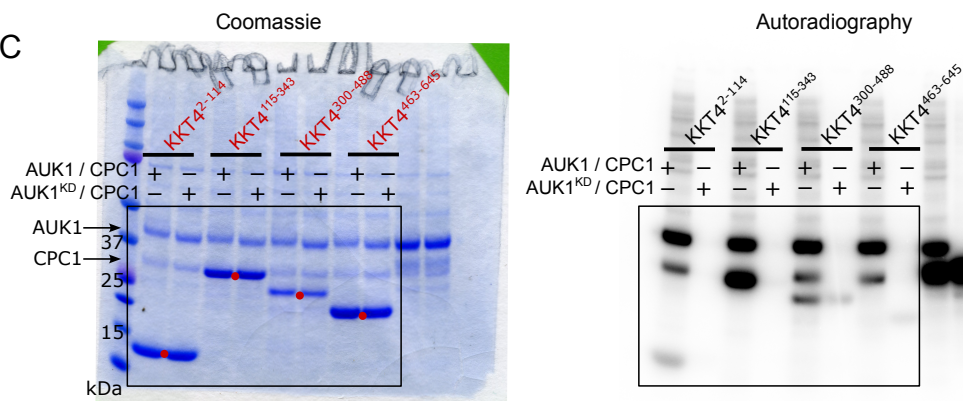

D

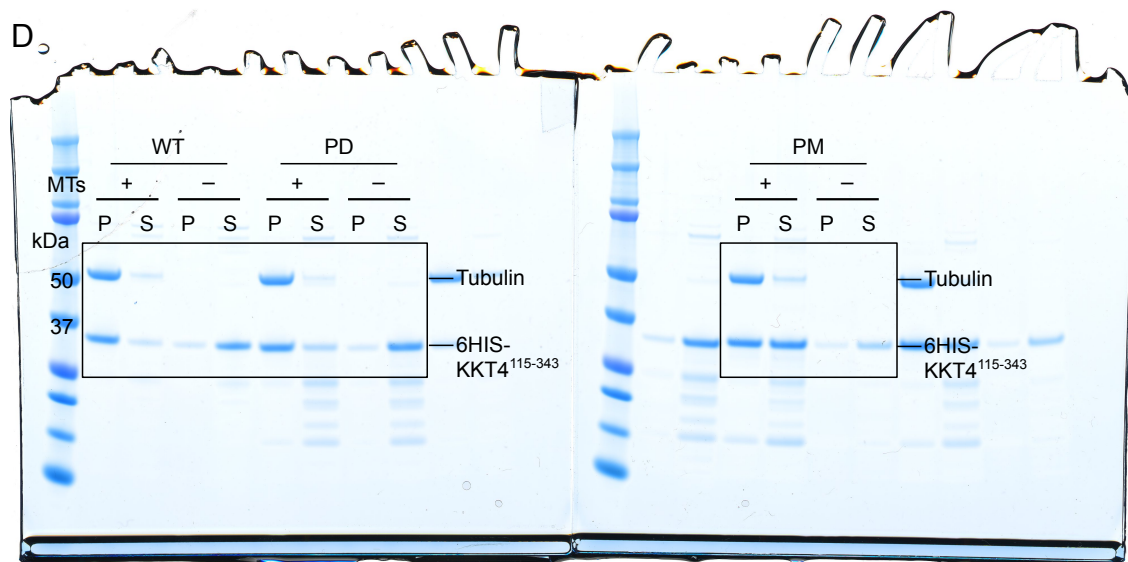

Supplement: SourceData FS3 — is the source file for Fig. S3. [file JCB_202401169_SourceDataFS3.pdf]

C

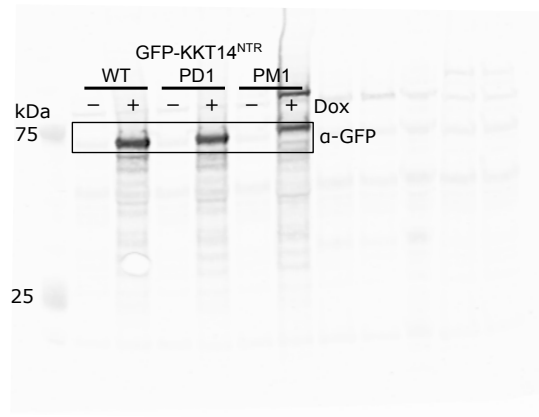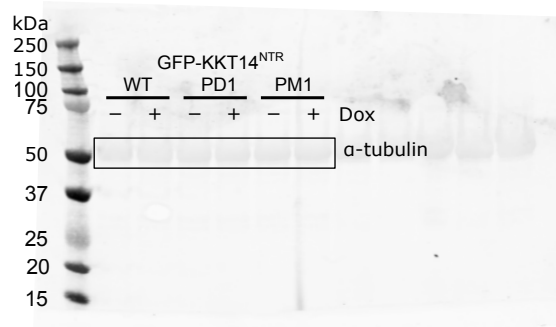

Supplement: SourceData FS4 — is the source file for Fig. S4. [file JCB_202401169_SourceDataFS4.pdf]
